# Ferroptosis‐Enhanced Immunotherapy with an Injectable Dextran‐Chitosan Hydrogel for the Treatment of Malignant Ascites in Hepatocellular Carcinoma

**DOI:** 10.1002/advs.202300517

**Published:** 2023-05-03

**Authors:** Jingshu Meng, Xiao Yang, Jing Huang, Zhan Tuo, Yan Hu, Zhiyun Liao, Yu Tian, Suke Deng, Yue Deng, Zhiyuan Zhou, Jonathan F. Lovell, Honglin Jin, Yang Liu, Kunyu Yang

**Affiliations:** ^1^ Cancer Center Union Hospital Tongji Medical College Huazhong University of Science and Technology Wuhan 430022 P. R. China; ^2^ College of Biomedicine and Health and College of Life Science and Technology Huazhong Agricultural University Wuhan 430070 P. R. China; ^3^ Department of Chemical and Biological Engineering University at Buffalo State University of New York Buffalo NY 14260 USA; ^4^ Key Laboratory of Polymer Ecomaterials Changchun Institute of Applied Chemistry Chinese Academy of Sciences 5625 Renmin Street Changchun 130022 P. R. China

**Keywords:** cancer immunotherapy, ferroptosis, hydrogel, malignant ascites, tumor microenvironment

## Abstract

Malignant ascites in advanced hepatocellular carcinoma (HCC) is a complex clinical problem that lacks effective treatments. Due to the insensitivity of advanced HCC cells to traditional chemotherapies, low drug accumulation, and limited drug residence time in the peritoneal cavity, the therapeutic effects of malignant ascites in HCC are not satisfactory. In this study, an injectable hydrogel drug delivery system based on chitosan hydrochloride and oxidized dextran (CH‐OD) is designed to load sulfasalazine (SSZ), an FDA‐approved drug with ferroptosis‐inducing ability, for effective tumor‐killing and activation of anti‐tumor immunity. Compared to free SSZ, SSZ‐loaded CH‐OD (CH‐OD‐SSZ) hydrogel exhibits greater cytotoxicity and induces higher levels of immunogenic ferroptosis. In the preclinical model of hepatoma ascites, intraperitoneal administration of CH‐OD‐SSZ hydrogel can significantly suppress tumor progression and improve the immune landscape. Both in vitro and in vivo, CH‐OD‐SSZ hydrogel induces the repolarization of macrophages to an M1‐like phenotype and promotes the maturation and activation of dendritic cells. Combination treatment with CH‐OD‐SSZ hydrogel and anti‐programmed cell death protein 1 (PD‐1) immunotherapy achieves more than 50% ascites regression and generates long‐term immune memory. Collectively, CH‐OD‐SSZ hydrogel exhibits promising therapeutic potential in the treatment of peritoneal dissemination and malignant ascites in advanced HCC, especially when combined with anti‐PD‐1 immunotherapy.

## Graphical abstract

This study introduces injectable chitosan hydrochloride‐oxidized dextran hydrogel loaded with sulfasalazine (CH‐OD‐SSZ) for sustained drug delivery and ferroptosis inducing in a malignant ascites model of hepatocellular carcinoma. CH‐OD‐SSZ possesses both a direct tumor‐killing effect and immune‐stimulating activity to repolarize M2 macrophages and promote the activation of dendritic cells, efficiently suppressing the ascites progression and enhancing the anti‐PD‐1 immunotherapy in vivo.

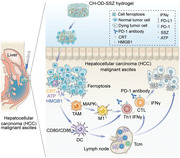

## 1 Introduction

Hepatocellular carcinoma (HCC) represents a significant public health issue globally, particularly in East Asia and Africa, and is a leading cause of cancer‐related death.^[^ ^1^, ^2^ ^]^ Since 2015, significant advances have been made in the management of HCC, especially in the development of systematic therapies, which have led to marked improvements in the overall survival and life quality of patients.^[^ ^3^ ^]^ However, existing strategies, including resection, transplantation, ablation, transarterial chemoembolization, or novel combination regimens such as immunotherapy and targeted therapy, are not suitable or effective for certain patients with malignant ascites caused by advanced HCC.^[^ ^2^, ^4^, ^5^, ^6^ ^]^ Broad resistance of advanced HCC to systematic therapies,^[^ ^7^, ^8^ ^]^ low accumulation of drugs, and limited drug residence time in the peritoneal cavity^[^ ^9^, ^10^ ^]^ often lead to suboptimal therapeutic effects. As malignant ascites have been observed in 23% of patients diagnosed with HCC and are considered as a signal of tumor progression and poor prognosis,^[^ ^11^, ^12^ ^]^ it is of great importance to seek new strategies for malignant ascites arising from HCC.

Ferroptosis is an iron‐dependent form of programmed cell death (PCD) triggered by overloaded membrane lipid peroxidation and decreased antioxidant activity.^[^ ^13^, ^14^ ^]^ In recent years, ferroptosis has emerged as an attractive target for cancer treatment, particularly for aggressive malignancies that are resistant to apoptosis induced by traditional therapies.^[^ ^13^, ^14^ ^]^ In general, tumor cells are more metabolically active than normal cells to meet their increased metabolic demands for rapid proliferation, which simultaneously exposes them to oxidative stress and a higher load of reactive oxygen species (ROS), resulting in a unique susceptibility to ferroptosis.^[^ ^15^, ^16^ ^]^ Studies have shown that therapy‐refractory tumor cells, especially those with high‐mesenchymal phenotypes, are enriched in polyunsaturated fatty acids and heavily depend on glutathione peroxidase 4 (GPX4) to reverse lipid peroxidation for survival.^[^ ^17^, ^18^ ^]^ Disruption of the cysteine and glutathione (GSH)‐GPX4 system could induce ferroptosis in such apoptosis‐resistant tumor cells. In a previous study, Wang et al. reported that interferon‐*γ* (IFN‐*γ*) produced by CD8^+^ cytotoxic T cells could enhance tumor cell ferroptosis through the inhibition of solute carrier family 7 member 11 (SLC7A11; a transporter subunit of system xc^−^) expression. A ferroptosis inducer cysteinase combined with immune checkpoint blockade could synergistically enhance the T cell‐mediated antitumor immune response.^[^ ^19^ ^]^ Of note, ferroptosis has been recently identified as a form of immunogenic cell death (ICD), as early ferroptotic tumor cells release damage‐associated molecular patterns (DAMPs) to promote antigen‐presenting cell (APC) maturation and subsequent priming of effector T cells.^[^ ^20^, ^21^, ^22^ ^]^ Such evidence suggests that targeting ferroptosis may be a promising strategy to overcome cancer therapy resistance and augment antitumor immunity.

Several ferroptosis inducers (FINs) have been proven to be effective in preclinical tumor models, including breast cancer,^[^ ^23^ ^]^ small cell lung cancer,^[^ ^24^ ^]^ melanoma,^[^ ^16^ ^]^ and renal cell carcinoma,^[^ ^25^ ^]^ but their application in HCC with malignant ascites has not been reported. Sulfasalazine (SSZ), an FDA‐approved first‐line drug for the treatment of rheumatoid arthritis and ulcerative colitis, has been shown to be effective in inducing ferroptosis in tumor cells by inhibiting SLC7A11.^[^ ^26^, ^27^ ^]^ However, the poor water solubility and metabolic stability of SSZ limit its further clinical application in tumor therapy.^[^ ^28^ ^]^ In vivo application of such small molecule drugs often requires repeated administration or high dosages to achieve a therapeutic effect, which can lower patients’ compliance and lead to extra adverse effects.

Currently, hydrogels have been widely explored and used in wound healing, drug delivery, tissue engineering, and other medical applications according to their variable physicochemical, biological, and structural characteristics.^[^ ^29^, ^30^ ^]^ Compared to local injection of free drugs or systemic administration, local drug delivery through hydrogels enables the quick forming of a drug reservoir at target disease sites and the release of drugs over an extended period.^[^ ^31^ ^]^ Polysaccharides, such as dextran and chitosan, are attractive hydrogel natural materials due to their excellent biodegradability, biocompatibility, and improved safety profile for clinical application compared to synthetic polymers.^[^ ^32^, ^33^ ^]^ The adjacent hydroxyl groups in dextran can be selectively oxidized to form aldehyde groups, which can react with amine‐ or hydrazide‐containing molecules via a Schiff‐base crosslinking.^[^ ^34^, ^35^ ^]^ The process is typically very fast and mild, providing great potential for preparing in situ formable or injectable hydrogels.^[^ ^36^ ^]^ In this study, we developed an injectable and in situ cross‐linked hydrogel system based on chitosan hydrochloride and oxidized dextran (CH‐OD), to deliver SSZ for localized therapy in HCC with malignant ascites. Intraperitoneal administration of SSZ‐loaded CH‐OD (CH‐OD‐SSZ) hydrogel could realize high levels of SSZ loading and localized and sustained release of SSZ in the peritoneal cavity. This effectively induced immunogenic ferroptosis and provoked a systemic antitumor immune response. Furthermore, CH‐OD‐SSZ hydrogel exhibited intrinsic immunostimulatory activity, which repolarized tumor‐associated macrophages (TAMs) into a pro‐inflammatory, tumor‐killing M1‐like phenotype, and promoted the maturation of dendritic cells (DCs). When combined with anti‐programmed cell death protein 1 (PD‐1) immunotherapy, the CH‐OD‐SSZ hydrogel system achieved a cure rate of 58% and induced durable immunological memory. Collectively, our results proved the therapeutic efficacy of CH‐OD‐SSZ‐mediated ferroptosis and immune activation, providing a promising strategy for the treatment of peritoneal dissemination and malignant ascites in advanced HCC.

## 2 Results

### 2.1 High Expression of SLC7A11 Correlates with Poor Prognosis in HCC

**Figure 1:**
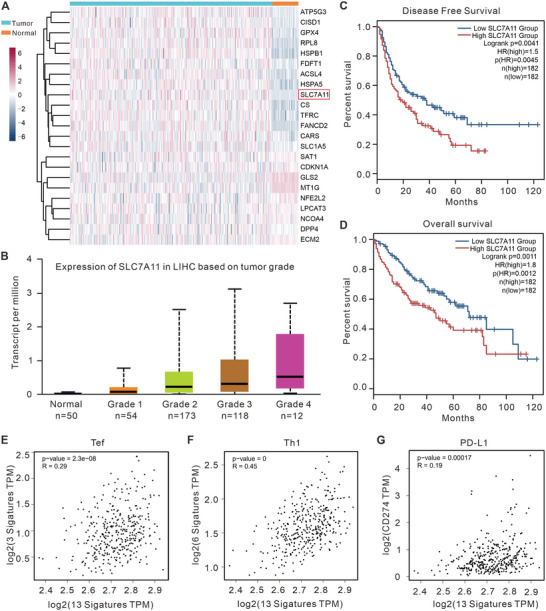
Ferroptosis‐related molecular patterns with distinct prognosis, T cell infiltration, and functions in HCC. A) Heatmap depicting expression levels of ferroptosis‐related genes in HCC and adjacent normal tissues. B) The mRNA level of SLC7A11 correlates with the histologic grade of HCC. C,D) The Kaplan–Meier curves for DFS (C) and OS (D) for patients with HCC and either high or low SLC7A11 expression. E–G) Regression analysis of the correlation between the ferroptosis‐related gene signature and (E) Teff cells infiltration, (F) Th1 cells infiltration, or (G) CD274 gene expression.

To evaluate the clinical significance of ferroptosis in HCC, the mRNA expression levels of 23 ferroptosis‐related genes^[^ ^37^ ^]^ in HCC and adjacent non‐tumorous samples from The Cancer Genome Atlas (TCGA) database were analyzed. As shown in **Figure** **1**A, the mRNA expression level of SLC7A11 was significantly elevated in HCC samples compared to adjacent normal liver tissues, and its expression increased with the histologic grade of HCC (Figure 1B). Kaplan–Meier survival analysis indicated that HCC patients with high SLC7A11 expression levels tended to have a shorter disease‐free survival (DFS) and overall survival (OS) compared to those with low SLC7A11 expression (Figure 1C,D). The above results indicate that elevated SLC7A11 expression levels are related to higher malignancy and poor prognosis in patients with HCC.

To investigate the correlation between ferroptosis and tumor immune status, we calculated single‐sample gene set enrichment analysis (ssGSEA) scores of various immune cell types based on gene expression data in HCC samples and analyzed their correlation with ferroptosis. As shown in Figure 1E–G, ferroptosis positive regulatory gene signatures were positively correlated with the infiltration of effector T (Teff) cells (R = 0.29), T helper type 1 (Th1) cells (R = 0.45), and programmed death‐ligand 1 (PD‐L1, also known as CD274; R = 0.19). These data suggest that the induction of ferroptosis may enhance the infiltration of T cells into the tumor microenvironment (TME) and augment the efficacy of immunotherapy in HCC treatment. Overall, these results suggest that elevated expression of SLC7A11 and ferroptosis evasion in tumor cells are inversely correlated with clinical prognosis and antitumor immunity in HCC.

### 2.2 Preparation and Characterization of CH‐OD‐SSZ Hydrogel

**Figure 2:**
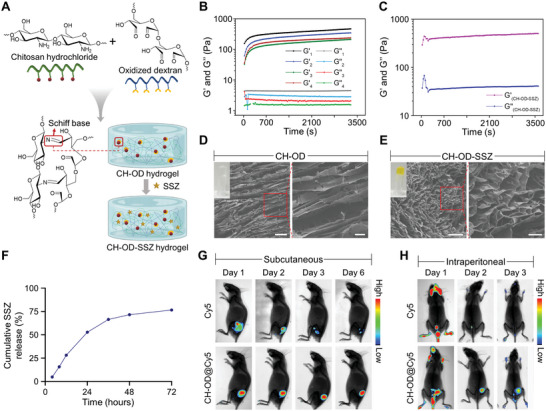
The construction of CH‐OD‐SSZ hydrogel and drug‐releasing behaviors. A) Schematic representation of Schiff's base reaction between CH and OD and CH‐OD‐SSZ hydrogel. B) Time evolution of storage modulus (*G'*) and loss modulus (*G“”*) for 3.5% (w/v) CH‐OD hydrogel at 37 °C with different feed ratios. The ratio of amino groups in CH to aldehyde groups in OD is 1.5:1 (Group 1), 1:1 (Group 2), 1:1.5 (Group 3), and 1:2 (Group 4). C) Time evolution of *G'* and *G“”* for CH‐OD‐SSZ hydrogel at 37 °C. D,E) Scanning electron microscope (SEM) images of the representative lyophilized CH‐OD hydrogel (D) and CH‐OD‐SSZ hydrogel (E) (Scale bar [left] = 20 µm, Scale bar [right] = 5 µm). Insets in the upper left corner are photographs of the two kinds of hydrogel. F) Release of SSZ from CH‐OD‐SSZ hydrogel loaded at 16 mg mL^−1^ in 0.1 m PBS solution in vitro. The amounts of SSZ released from hydrogel are normalized to initial amounts of SSZ loaded to each hydrogel formulation. The data are presented as the mean ± SEM of three independent experiments. G) Fluorescence images of mice taken on days 1, 2, 3, and 6 after subcutaneous injection of free Cy5 or CH‐OD@Cy5 hydrogel. H) Fluorescence images of mice taken on days 1, 2, and 3 after peritoneal injection of free Cy5 or CH‐OD@Cy5 hydrogel.

Due to the abnormally high expression of SLC7A11 in HCC, which is negatively correlated with antitumor immunity and clinical prognosis, inhibiting SLC7A11 and promoting ferroptosis may be a promising strategy for HCC treatment. To investigate the therapeutic effect of ferroptosis in HCC, we selected sulfasalazine, an inhibitor of SLC7A11/system xc^−^, as a ferroptosis‐inducing drug in our study and designed chitosan hydrochloride and oxidized dextran hydrogel to improve drug delivery and maintain effective drug concentration in the peritoneal cavity. To improve the solubility of chitosan, we prepared chitosan hydrochloride using a 60% ethanolic HCl solution. Next, the vicinal diol structure in dextran was oxidized by sodium periodate to obtain an aldehyde group. The successful synthesis of OD was confirmed by Fourier transform infrared spectrometry (FT‐IR, Figure S1A, Supporting Information). As shown in **Figure** **2**A, the amino group in CH could readily react with the aldehyde group in OD through Schiff's base, leading to the instantaneous formation of CH‐OD hydrogel within 5 s after mixing the two solutions. To construct an optimal network structure for drug loading, we tried four different ratios of CH and OD with a fixed final concentration of 3.5%. By regulating the feed ratio of CH and OD solutions, the ratio of the amino group to the aldehyde group in the hydrogel was either 1.5:1, 1:1, 1:1.5, or 1:2. We found that the hydrogel containing excess amino groups, namely the ratio of 1.5:1, possessed the largest storage modulus (*G'*) and loss modulus (*G“”*) (Figure 2B), and was therefore selected for the following experiments. Furthermore, the modulus of the polysaccharide‐based hydrogel hardly changed after SSZ loading (Figure 2C). As depicted in Figure 2D,E, there were obvious lamellar crosslinking structures in the interior of the hydrogel, and the addition of SSZ had no impact on the structure of the hydrogel. The similar modulus of CH‐OD and CH‐OD‐SSZ hydrogel as a function of frequency was also observed (Figure S1B, Supporting Information). The insets in the top left corner of Figure 2D,E showed the stable appearance of CH‐OD hydrogel before and after SSZ loading. When the shear rate was constant, the viscosity of the hydrogel increased rapidly and reached a steady state soon, indicating that it could quickly form a gel (Figure S2A, Supporting Information). The shear rate sweep showed that the viscosity of the hydrogel decreased with increasing shear rates, indicating its good injectability (Figure S2B, Supporting Information). The swelling ratio of lyophilized hydrogel in PBS first increased significantly, and then the rising trend became slower with the passage of soaking time until the final steady state was reached (Figure S2C, Supporting Information). About three‐quarters of loaded SSZ were gently released from CH‐OD hydrogel within 72 h in PBS at 37 °C in vitro (Figure 2F). The degradation behavior and biodegradability of hydrogels are important for the biosafety of materials and drug release. PBS containing protease K was used in vitro to simulate the in vivo degradation environment of the hydrogel. Compared with the plain PBS group, the additional addition of protease K significantly accelerated the degradation of hydrogel but generally maintained a slow degradation state (Figure S3A, Supporting Information). To observe the degradation of hydrogel in vivo, hydrogels were injected subcutaneously into the right flank of mice. The local skin at the injection site formed spherical ridges without spreading to the surrounding area, indicating that the hydrogels gathered at the injection site in a gelatinous shape. The degradation of the hydrogels was observed regularly. With the extension of time, the spherical uplift formed at the injection site gradually became smaller, and the hydrogels were basically completely degraded in about 21 days (Figure S3B, Supporting Information). These results indicate that hydrogels can be degraded slowly in vivo, suggesting that they own sustained‐release properties and good biosafety. In addition, we characterized the drug‐releasing behavior of CH‐OD‐SSZ hydrogel after subcutaneous or intraperitoneal administration in vivo. As shown in Figure 2G, Cy5 was used for fluorescence observation, and we found that Cy5 loaded in the CH‐OD hydrogel produced a strong fluorescence signal even after six days of injection. In comparison, the signal of free Cy5 decreased steeply within three days. In line with these results, the CH‐OD hydrogel system also improved drug retention in the peritoneal cavity, which prolonged the residence time from one day of free Cy5 to at least three days of CH‐OD‐Cy5 (Figure 2H). In the context of malignant ascites, CH‐OD hydrogel provided a prolonged presence of SSZ for a minimum of six days (Figure S3C, Supporting Information). Therefore, CH‐OD‐SSZ hydrogel can maintain the effective concentration of drugs in the TME and achieve a more effective curative effect.

### 2.3 In Vitro Antitumor Mechanism and Immunogenic Death Mode

**Figure 3:**
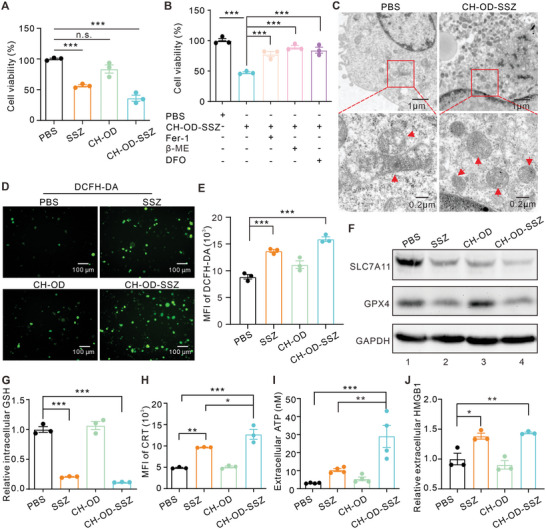
In vitro cytotoxicity and mechanism of cell death induced by CH‐OD‐SSZ hydrogel. A) Cell viability of H22 cells treated with PBS, SSZ, CH‐OD, or CH‐OD‐SSZ hydrogel. B) Cell viability of CH‐OD‐SSZ‐incubated H22 cells in the presence of variant ferroptosis inhibitors. C) TEM images of H22 cells treated with PBS and CH‐OD‐SSZ hydrogel for 48 h. Red arrowheads indicate mitochondria. D) Representative fluorescence images of H22 cells treated with PBS, SSZ, CH‐OD, or CH‐OD‐SSZ hydrogel, and intracellular ROS production stained with DCFH‐DA (green) probe. E) ROS production was analyzed by flow cytometry using DCFH‐DA in different treatment groups. F) Protein expression levels of SLC7A11 and GPX4 in H22 cells treated with PBS, SSZ, CH‐OD, or CH‐OD‐SSZ hydrogel for 48 h were analyzed by Western blotting. G) Flow cytometry analysis of intracellular GSH levels in H22 cells treated with PBS, SSZ, CH‐OD, or CH‐OD‐SSZ hydrogel for 48 h. H) Flow cytometric analysis of CRT expression. I) ATP levels in the culture supernatant of H22 cells were evaluated by luciferin‐based ATP assay. J) ELISA assay for the detection of HMGB1 in the culture supernatant of H22 cells. The indicated results represent the mean ± SEM of three independent experiments. *P < 0.05, **P < 0.01, ***P < 0.001, n.s., not significant.

Next, we verified whether the designed CH‐OD‐SSZ hydrogel could kill tumor cells and induce ferroptosis as expected. As shown in **Figure** **3**A and Figure S4A–C, Supporting Information, CCK‐8 assays and colony formation analysis showed that both SSZ and CH‐OD‐SSZ hydrogel could effectively inhibit the growth of H22 cells, while CH‐OD hydrogel exhibited no direct effects on cell growth. Three ferroptosis inhibitors, including ferrostatin‐1 (Fer‐1), *β*‐mercaptoethanol (*β*‐ME), and iron chelator deferoxamine (DFO), could rescue the growth inhibition of H22 cells induced by CH‐OD‐SSZ hydrogel (Figure 3B). To further illustrate the characteristics of cell death caused by CH‐OD‐SSZ hydrogel, we assessed the morphological changes of CH‐OD‐SSZ‐treated cells by transmission electron microscope (TEM). Compared with the control group, CH‐OD‐SSZ hydrogel‐treated H22 cells exhibited typical morphological features of ferroptosis,^[^ ^38^ ^]^ such as shrunken mitochondria, disappearance of mitochondrial cristae, and outer mitochondrial membrane rupture (Figure 3C). These results suggest that CH‐OD‐SSZ hydrogel‐induced inhibition of tumor cell growth is achieved by inducing tumor cell ferroptosis. Due to the collapse of cellular redox homeostasis during ferroptosis, we measured the intracellular and lipid ROS levels as well as the glutathione contents of H22 cells treated with PBS (control), SSZ, CH‐OD, or CH‐OD‐SSZ hydrogel. H22 cells treated with SSZ and CH‐OD‐SSZ hydrogel exhibited stronger fluorescence intensity after incubation with ROS probes *2′,7′*‐dichlorodihydrofluorescein diacetate (DCFH‐DA), and 4,4‐difluoro‐5‐(4‐phenyl‐1,3‐butadienyl)‐4‐bora‐3a,4a‐diaza‐s‐indacene‐3‐undecanoic acid (BODIPY 581/591 C11) (Figure 3D, Figure S4D, Supporting Information). The quantification of intracellular ROS measured by flow cytometry was consistent with the fluorescence results (Figure 3E). Moreover, intracellular GSH contents in the SSZ and CH‐OD‐SSZ hydrogel group were 80% and 89% lower, respectively, than those in the PBS group (Figure 3G). To further elucidate the underlying mechanism, we analyzed the protein expression levels of SLC7A11 and GPX4 by Western blotting. As shown in Figure 3F, SSZ and CH‐OD‐SSZ hydrogel treatment significantly down‐regulated SLC7A11 and GPX4 in H22 cells. Collectively, our results demonstrated that CH‐OD‐SSZ hydrogel could inhibit SLC7A11/system xc^−^ to induce the depletion of intracellular GSH and simultaneously inhibit GPX4 expression to promote the ferroptosis of tumor cells.

Recent studies have preliminarily identified ferroptosis as an immunogenic cell death, characterized by the release or exposure of DAMPs.^[^ ^21^, ^39^ ^]^ To validate this, we examined the release of DAMPs from H22 cells treated with PBS, SSZ, CH‐OD, or CH‐OD‐SSZ hydrogel. As shown in Figure 3H,I, the surface expression of calreticulin (CRT) and the release of cellular adenosine triphosphate (ATP) were significantly increased after treatment with SSZ or CH‐OD‐SSZ hydrogel. H22 cells treated with CH‐OD‐SSZ hydrogel exhibited a 2.6‐fold increase in extracellular CRT levels and a 9.3‐fold increase in ATP release compared to those treated with PBS. Consistently, treatment with SSZ and CH‐OD‐SSZ hydrogel significantly elevated the release of high mobility group box‐1 protein (HMGB1), another key indicator of ICD (Figure 3J). Overall, these findings indicate that the CH‐OD‐SSZ hydrogel efficiently induces ICD in tumor cells.

### 2.4 In Vitro Immune Activation Effect

**Figure 4:**
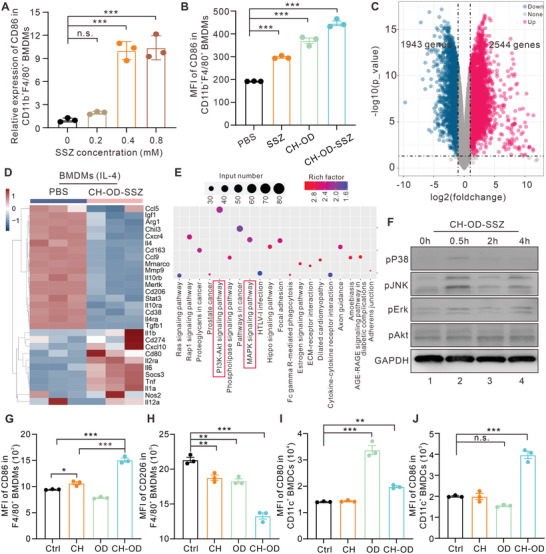
CH‐OD‐SSZ‐induced M1 polarization of macrophages in vitro. A,B) The proportion of M1‐like macrophages in BMDMs was analyzed by flow cytometry. BMDMs were treated with different concentrations of SSZ (A) or PBS, SSZ, CH‐OD, and CH‐OD‐SSZ hydrogel (B) for 24 h. C–E) RNA‐seq analysis of BMDMs incubated with PBS or CH‐OD‐SSZ hydrogel for 24 h. (C) Volcano plot showing the up‐regulated, down‐regulated or insignificantly differentially expressed genes. (D) Heatmap illustrating the fold changes of M1‐ and M2‐macrophage‐related gene sets, as indicated. (E) KEGG pathway enrichment analysis reveals the activation of PI3K/Akt and MAPK signaling pathway components as indicated in BMDMs treated with CH‐OD‐SSZ hydrogel. F) Protein expression levels of p‐p38, p‐JNK, p‐ERK, and p‐Akt in BMDMs treated with CH‐OD‐SSZ hydrogel at the indicated time points were analyzed by Western blotting. G,H) Flow cytometry analysis of the expression of CD86 (M1 marker) and CD206 (M2 marker) in BMDMs. I,J) Flow cytometry analysis of the expression of CD80 and CD86 in BMDCs. BMDMs or BMDCs were treated with PBS, CH, OD, or CH‐OD hydrogel for 24 h. The indicated results represent the mean ± SEM of three independent experiments. *P < 0.05, **P < 0.01, ***P < 0.001 analyzed by one‐way ANOVA.

Considering the up‐regulation of ICD markers induced by CH‐OD‐SSZ hydrogel, we subsequently investigated whether CH‐OD‐SSZ hydrogel could exert an immune activation effect on macrophages or DCs in vitro. In the TME, TAMs usually exhibit an anti‐inflammatory and immunosuppressive M2‐like phenotype, which is associated with immune evasion and tumor progression.^[^ ^40^ ^]^ To explore the effect of CH‐OD‐SSZ hydrogel on macrophage polarization, mouse bone marrow‐derived macrophages (BMDMs) were isolated and polarized to M2‐like phenotype using IL‐4, and then exposed to PBS, SSZ, CH‐OD, or CH‐OD‐SSZ hydrogel. As shown in **Figure** **4**A, SSZ increased the expression of CD86 (M1 marker) in a concentration‐dependent manner. Moreover, SSZ, CH‐OD, and CH‐OD‐SSZ hydrogel all significantly promoted the polarization of BMDMs to an M1‐like phenotype, as indicated by the increased expression of CD86, and CH‐OD‐SSZ hydrogel showed the strongest effect among the three groups (Figure 4B, Figure S5A, Supporting Information). Additionally, we examined whether CH‐OD‐SSZ hydrogel could exhibit cytotoxic effects on BMDMs. As shown in Figure S5B,C, Supporting Information, despite its effectiveness in killing H22 cells, CH‐OD‐SSZ hydrogel did not significantly affect the viability of macrophages at the same concentration.

To explore the potential molecular mechanism underlying the phenotype reprogramming of macrophages induced by CH‐OD‐SSZ hydrogel, we performed RNA sequencing (RNA‐seq) analysis on BMDMs that were either treated with PBS or CH‐OD‐SSZ hydrogel and identified 2187 differentially expressed genes (DEGs, Figure 4C). Compared with the control group, CH‐OD‐SSZ‐treated BMDMs had higher levels of M1‐related transcripts and lower levels of M2‐related transcripts, suggesting a shift towards an M1‐like phenotype. Specifically, as shown in Figure 4D, M1‐related genes, including *Nos2*, *Il1b*, *Il6*, *Il12a*, *Cd80*, and *Tnf*, were significantly up‐regulated, while M2‐related genes, including *Arg1*, *Il4*, *Tgfb1*, *Chil3*, *Cd206*, *Cd163*, and *Mertk*, were significantly down‐regulated in CH‐OD‐SSZ‐treated BMDMs. To further access the underlying signaling pathways, we performed Kyoto Encyclopedia of Genes and Genomes (KEGG) analysis on the DEGs. KEGG analysis revealed that the M1‐related mitogen‐activated protein kinase (MAPK) and phosphatidylinositide 3‐kinase (PI3K)/Akt signaling pathways were enriched in CH‐OD‐SSZ‐treated BMDMs compared with the control group (Figure 4E). Since both MAPK and PI3K/Akt pathways have been reported to play an essential role in macrophage polarization,^[^ ^41^, ^42^ ^]^ we further validated the phosphorylation levels of three major proteins (p‐p38, p‐JNK, and p‐ERK) of the MAPK pathway, as well as p‐Akt in CH‐OD‐SSZ‐treated BMDMs by Western blotting. As shown in Figure 4F, CH‐OD‐SSZ hydrogel treatment significantly upregulated the protein levels of p‐p38, p‐ERK1/2, and p‐JNK but had no effect on p‐AKT. Collectively, these findings suggest that CH‐OD‐SSZ hydrogel reprograms macrophages from a protumor M2‐like phenotype to an antitumor M1‐like phenotype via the activation of the MAPK signaling pathway.

Furthermore, we explored whether CH‐OD‐SSZ hydrogel could promote the maturation and activation of DCs in vitro. As specialized APCs, DCs play a pivotal role in processing and presenting tumor antigens to prime T cells. For the DC stimulation experiment, H22 cells pretreated with PBS, SSZ, CH‐OD, or CH‐OD‐SSZ hydrogel were harvested and co‐cultured with bone marrow‐derived dendritic cells (BMDCs, Figure S6A, Supporting Information). Then, the expression of the costimulatory molecules CD80 and CD86 in BMDCs was analyzed by flow cytometry. As shown in Figure S6B,C, Supporting Information, CH‐OD‐SSZ‐treated H22 cells significantly increased the expression of CD80 and CD86 in BMDCs, indicating that CH‐OD‐SSZ hydrogel could promote the maturation and activation of DCs. In addition, considering the immune activation effect of the CH‐OD hydrogel itself on macrophages and DCs, we conducted an additional evaluation to determine the separate contributions of CH and OD polysaccharides. As shown in Figure 4G–J, both CH and OD significantly decreased the expression of CD206 (M2 marker) in BMDMs, and OD exhibited a remarkable ability to stimulate the expression of CD80 in BMDCs, indicating their intrinsic immune‐stimulating capability.

### 2.5 In Vivo Antitumor Effects and Immune Activation of the CH‐OD‐SSZ Hydrogel in HCC‐Related Malignant Ascites

**Figure 5:**
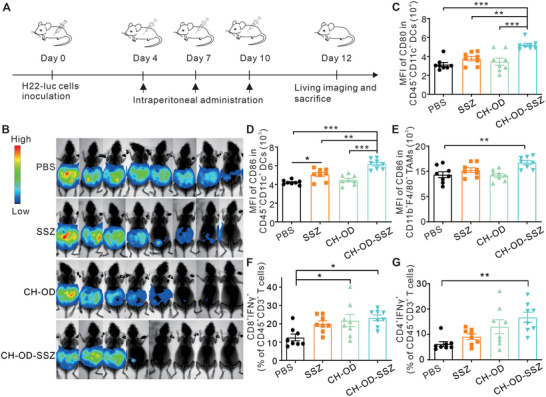
In vivo therapeutic effects and immune activation of CH‐OD‐SSZ hydrogel. A) Animal experiment outline for the treatment of mice intraperitoneally injected with H22‐luc hepatoma cells. B) In vivo bioluminescence images of H22‐luc hepatoma ascites mice under different treatment conditions. C‐G) Tumor immune microenvironment of ascites was detected by flow cytometry (n = 8 mice per group). (C, D) The expression of CD80 (C) and CD86 (D) in DCs (CD45^+^CD11c^+^). (E) The expression of CD86 in TAMs (CD45^+^CD11b^+^F4/80^+^). (F, G) The percentage of CTLs (CD8^+^IFN*γ* ^+^, (F)) and Th1 cells (CD4^+^IFN*γ* ^+^, (G)) in T cells (CD45^+^CD3^+^). *P < 0.05, **P < 0.01, ***P < 0.001 analyzed by one‐way ANOVA.

As noted above, the CH‐OD‐SSZ hydrogel could repolarize macrophages towards a pro‐inflammatory M1‐like phenotype and promote the maturation and activation of DCs in vitro. To further evaluate the therapeutic potential of CH‐OD‐SSZ hydrogel in vivo, we established a malignant ascites mouse model by intraperitoneal injection of luciferase‐tagged H22 cells (H22‐luc) into BALB/c mice, and subsequently traced the development of malignant ascites by monitoring the bioluminescence signals of H22‐luc cells. The formation of malignant ascites was confirmed by bioluminescence imaging three days after inoculation, and the mice that successfully formed ascites were randomly divided into four groups and treated with PBS, SSZ, CH‐OD or CH‐OD‐SSZ hydrogel, respectively (**Figure** **5**A). As shown in Figure 5B, intraperitoneal injection of SSZ, CH‐OD, and CH‐OD‐SSZ hydrogel could reduce the tumor burden to varying degrees compared with the PBS group. Among them, CH‐OD‐SSZ hydrogel exhibited the best antitumor effects. Survival analysis showed that the CH‐OD‐SSZ hydrogel group had a significantly prolonged survival time than the other three groups, with a complete response rate of 25% (3/12) and no recurrence (Figure S7A, Supporting Information). These results indicate that CH‐OD‐SSZ hydrogel has significant therapeutic effects against HCC ascites in vivo and provides survival benefits.

To investigate the effects of different treatments on the tumor immune microenvironment (TIME), the phenotype of immune cells in the peritoneal lavage of mice was analyzed by flow cytometry. Gating strategies are indicated in Figure S8, Supporting Information. As shown in Figure 5C,D, the expression of CD80 and CD86 in DCs (CD11c^+^) were significantly increased in the CH‐OD‐SSZ hydrogel group, indicating that CH‐OD‐SSZ hydrogel treatment could promote the activation and maturation of DCs in the ascites. For macrophages, the mean fluorescence intensity (MFI) of CD86 in TAMs (CD11b^+^F4/80^+^) was significantly elevated in the CH‐OD‐SSZ hydrogel group (Figure 5E). Concurrently, we observed an increase in the proportion of cytotoxic T lymphocytes (CTLs) (CD8^+^IFN*γ* ^+^) and Th1 cells(CD4^+^IFN*γ* ^+^) in the CH‐OD‐SSZ group (Figure 5F,G), strongly suggesting the reshaping of the TIME and the augmentation of antitumor immunity. While SSZ exhibited an effective cytotoxic effect on H22 cells, the cell viability of DCs and T cells in the TIME was likely not affected (Figure S7B,C, Supporting Information). The proportion of Foxp3^+^ T regulatory cells was not statistically different between treated groups (Figure S7D, Supporting Information).

### 2.6 Synergistic Therapeutic and Long‐Term Immune Memory Effects Combined with Anti‐PD‐1 Immunotherapy

**Figure 6:**
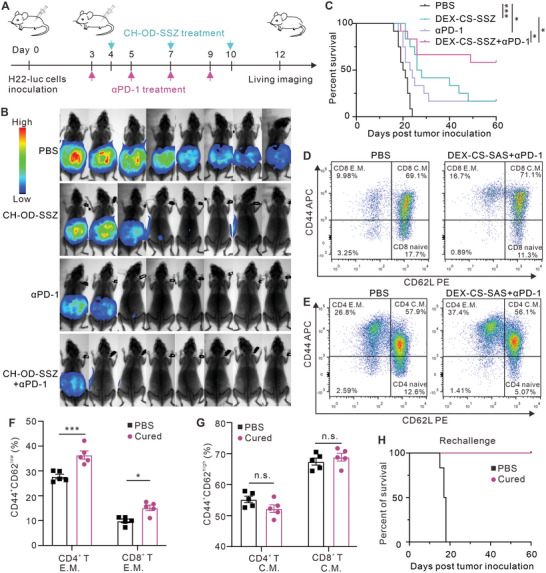
Combination therapy with CH‐OD‐SSZ hydrogel and anti‐PD‐1 in mice with malignant ascites. A) Animal experiment outline for CH‐OD‐SSZ hydrogel combined with anti‐PD‐1 in the treatment of mice with intraperitoneally injected H22‐luc hepatoma ascites. B) Representative bioluminescence images of H22‐luc hepatoma ascites mice under different treatment conditions. C) Kaplan–Meier survival analysis of H22‐luc hepatoma ascites mice that were treated with PBS, CH‐OD‐SSZ hydrogel, anti‐PD‐1, or combination therapy (n = 12 per group). D,E) Gating strategy of flow cytometry for Tem (CD44^high^CD62^low^) and Tcm (CD44^high^CD62L^high^) in the spleen. F,G) Proportions of Tem (F) and Tcm (G) in the spleen were analyzed by flow cytometry at day 60 (n = 5 per group). H) Kaplan–Meier survival analysis of cured mice that were intraperitoneally rechallenged with H22‐luc cells (n = 6 per group). *P < 0.05, ***P < 0.001, n.s., not significant.

As indicated above, CH‐OD‐SSZ hydrogel could induce ferroptosis in H22 cells, causing ICD and immune activation. The SSZ‐induced ferroptosis also significantly upregulated the expression of PD‐L1 on the surface of H22 cells (Figure S9A,B, Supporting Information). Additionally, the results of RNA‐seq and flow cytometry analysis showed that CH‐OD‐SSZ hydrogel could lead to a significant upregulation of PD‐L1 expression on macrophages (Figure 4D, Figure S9C, Supporting Information). The above results prompted our further investigation into the synergistic effect of CH‐OD‐SSZ hydrogel in combination with anti‐PD‐1 monoclonal antibody (mAb) (**Figure** **6**A). As shown in Figure 6B,C, the combination of CH‐OD‐SSZ hydrogel and anti‐PD‐1 significantly reduced tumor burden and prolonged survival time in comparison to CH‐OD‐SSZ hydrogel or anti‐PD‐1 alone. The combination treatment cured 58% (7/12) of the mice and achieved long‐term survival. Histopathological hematoxylin and eosin (H&E) staining of major organs (heart, liver, spleen, lung, and kidney) showed no observable pathological changes (Figure S10, Supporting Information), which proved the satisfactory biosafety of CH‐OD‐SZZ for in vivo treatment.

The formation of immunological memory is a hallmark of the adaptive antitumor immune response. This memory can provide long‐term suppression of tumor recurrence and metastasis. Therefore, we further assessed whether mice cured by the combination treatment with CH‐OD‐SSZ hydrogel and anti‐PD‐1 had acquired immunological memory. For this experiment, five H22 tumor‐bearing mice cured by the combination treatment were analyzed for the presence of memory T cells in the spleen at 60 days after initial treatment. CD3^+^CD44^high^CD62L^low^ cells are denoted as T effector memory (Tem) cells; and CD3^+^CD44^high^CD62L^high^ cells are denoted as T central memory (Tcm) cells (Figure 6D,E). In both the CD4 and CD8 compartments, the percentage of Tem in the cured mice was significantly higher than that of control mice, while the percentage of Tcm exhibited no observable changes (Figure 6F,G). The other cured mice were rechallenged with H22‐luc cells and healthy mice of the same age were challenged in parallel as a control group. We observed that all cured mice completely rejected the growth of rechallenged tumor cells and achieved long‐term survival, while the control mice quickly formed malignant ascites and eventually died (Figure 6H). Collectively, these results demonstrate that the combination treatment with CH‐OD‐SSZ hydrogel and anti‐PD‐1 has a synergistic antitumor effect and can induce protective immunological memory.

## 3 Discussion

Recent studies have demonstrated that ferroptosis is a form of ICD, characterized by the release or exposure of tumor‐associated antigens and DAMPs.^[^ ^20^, ^21^ ^]^ The induction of ICD in tumor therapy not only directly kills tumor cells but also induces systematic antitumor immune responses.^[^ ^39^ ^]^ Such strategies to elicit vaccination‐like effects are widely used to reprogram the TIME and synergize with immunotherapies to improve therapeutic efficiency and response rates.^[^ ^43^ ^]^ To take advantage of the potential benefits of ferroptosis‐induced ICD, we developed an in situ self‐gellable hydrogel for the local delivery of the ferroptosis inducer SSZ. We showed that loading SSZ into the CH‐OD hydrogel preserved its bioactivity for the induction of ferroptosis and enabled sustained local release at tumor sites upon intraperitoneal injection. In vitro, CH‐OD‐SSZ offered an SSZ‐based direct killing effect on tumor cells, which could be reversed by inhibitors of ferroptosis. CH‐OD‐SSZ‐treated tumor cells exhibited an overload of lipid ROS and depletion of GSH, as well as typical shrunken mitochondria. Additionally, we showed that CH‐OD‐SSZ could trigger ICD in tumor cells and increase the expression of DAMPs, which induced DC maturation in the coculture model.

TAMs are essential components of the TME and are closely involved in tumor progression by promoting metastasis, angiogenesis, and immunosuppression.^[^ ^44^ ^]^ Macrophages can be classified into two distinct phenotypes, classically activated (M1) or alternatively activated (M2) macrophages. Repolarization of TAMs towards a tumor‐inhibiting M1‐like phenotype or ablation of a protumor M2‐like phenotype is a promising strategy for cancer therapy. In HCC, tumor cells can interact with TAMs via a paracrine Wnt/*β*‐catenin signaling loop and promote M2 polarization, reinforcing a malignant phenotype.^[^ ^45^ ^]^ Compared to M1 macrophages, M2 macrophages are more susceptible to pharmacologically induced ferroptosis due to their lack of inducible nitric oxide synthase (iNOS)/NO•‐enrichment.^[^ ^46^ ^]^ Iron overload can lead to increased levels of M1 markers such as IL‐1*β*, IL‐6, TNF‐*α*, and iNOS, and decreased M2 makers such as Arg1 and KLF4, promoting polarization towards an M1‐like phenotype.^[^ ^47^ ^]^ In our study, we found that CH‐OD‐SSZ hydrogel could directly repolarize macrophages towards an antitumor M1‐like phenotype. RNA sequencing analysis and Western blotting identified activation of the MAPK signaling pathway as a critical molecular mechanism of CH‐OD‐SSZ‐mediated TAM repolarization. A previous study suggested that ferroptotic tumor cells are efficiently engulfed by macrophages via oxygenated phosphatidylethanolamine SAPE‐OOH that binds to the TLR2 on macrophages.^[^ ^48^ ^]^ ATP, HMGB1, and pro‐inflammatory cytokines released by ferroptotic cells can promote the recruitment and activation of macrophages towards an M1‐like phenotype.^[^ ^49^ ^]^ In another study, KRAS protein released by ferroptotic pancreatic tumor cells led to a protumor M2‐like phenotype, indicating that the immunogenic effects of ferroptosis depend on the context of TME and the type of DAMPs.^[^ ^50^ ^]^ CH‐OD hydrogel itself also displayed the ability to promote the M1 polarization in macrophages. The result is consistent with previous studies showing that chitosan is a potent activator of the NLRP3 inflammasome in macrophages,^[^ ^51^, ^52^ ^]^ and can promote the release of pro‐inflammatory cytokines interleukin‐1b, tumor necrosis factor, and chemokines associated with M1 macrophage polarization.^[^ ^53^, ^54^ ^]^

Malignant ascites represent a significant clinical challenge in the management of advanced HCC. Large‐volume paracentesis is the current standard of care for malignant ascites, which provides temporary symptom relief but requires frequently repeated operations and may lead to minor or severe complications such as peritoneal adhesions, peritonitis, hypovolemia, and protein loss.^[^ ^55^, ^56^ ^]^ Diuretics are typically ineffective and evidence for their use is weak.^[^ ^57^ ^]^ Furthermore, the presence of peritoneal metastases and ascites has been reported to be associated with extremely poor survival outcomes and high rates of primary resistance to ICI treatment due to a pervasive immunosuppressive microenvironment.^[^ ^58^, ^59^ ^]^ Several studies have attempted to establish engineered nanoparticles for the repolarization of TAMs or direct cytotoxicity of tumor cells in mouse models of malignant ascites.^[^ ^59^, ^60^, ^61^ ^]^ However, therapeutic regimens often required consecutive daily intraperitoneal administration for 1–2 weeks and were nearly incurable. Importantly, while frequent administration is essential to maintain high effective concentrations in the peritoneal cavity, it could lead to extra pain and infection risk for patients.^[^ ^62^ ^]^ Therefore, it is imperative to develop novel intraperitoneal drugs and delivery platforms. In our study, CH‐OD‐SSZ hydrogel potently improved drug retention in the peritoneal cavity and exhibited a satisfactory therapeutic effect when administered every three days for a total of three times in hepatoma ascites models. The intra‐abdominal environment with excess fluid accumulation is conducive to the degradation behavior and drug release of CH‐OD‐SSZ hydrogel, as well as the diffuse distribution of SSZ throughout the entire tumor environment. Combining CH‐OD‐SSZ hydrogel with anti‐PD‐1 treatment significantly prolonged survival, conferred a cure rate of over 50%, and generated long‐term protective immune memory against tumor rechallenge in cured mice. The ability of the CH‐OD‐SSZ hydrogel to remain in situ for extended periods of time allows for continuous regulation of tumor progression, mitigating the potential for repeated episodes of malignant ascites and the need for frequent administration, providing a promising strategy for the treatment of peritoneal dissemination and malignant ascites in advanced HCC.

Biomaterials and nanotechnology, including hydrogels, are being utilized to improve the targeted delivery, biodistribution, and release kinetics of the therapeutic cargoes to enhance their efficacy and safety.^[^ ^30^, ^63^ ^]^ In most cases, the hydrogel scaffold itself only acts as a container to provide sustained release of loaded antitumor agents without any additional antitumor or immunomodulatory functions.^[^ ^64^ ^]^ Previous articles on chitosan‐dextran hydrogel have mainly focused on its application in wound healing and tissue regeneration.^[^ ^35^, ^65^, ^66^ ^]^ Herein, we explored the potential of using chitosan‐dextran hydrogel as a localized drug delivery system for drugs with low water solubility and metabolic stability, such as sulfasalazine, to achieve sustained release and improved bioavailability at the site of administration for cancer treatment. CH and OD could instantaneously form CH‐OD hydrogel within 5 s after mixing, and we adjusted the ratio of amino groups in CH to aldehyde groups in OD based on *G'* and *G“”* modulus to optimize the network structure for drug loading. Natural polysaccharides are highly biocompatible, biodegradable, and suitable for the fabrication of hydrogels for biomedical applications compared to synthetic polymers.^[^ ^67^, ^68^ ^]^ Native chitosan is only soluble in diluted acid, which greatly limits its processibility and applications. To address this issue, we introduced hydrochloric acid into the structure of chitosan to form amphoteric chitosan hydrochloride, which dissolves easily in water. Additionally, dextran was oxidized with periodate to introduce multiple aldehyde groups into its backbone, making it an effective macromolecular crosslinker for polymers abundant in amino groups to formulate hydrogels. The CD‐OD hydrogel provides a cross‐linked 3D network that serves as an effective drug depot to facilitate local drug delivery and sustained release. Notably, the CD‐OD hydrogel was found to be a potent adjuvant biomaterial with the intrinsic immunostimulatory capability to promote the maturation of DCs and repolarization of macrophages towards an M1 phenotype, comparable in potency to SSZ‐induced ICD. Previous studies have demonstrated that chitosan can activate macrophages,^[^ ^69^ ^]^ and induce cytokine secretion from natural killer (NK) cells.^[^ ^70^ ^]^ Carroll et al. recently reported that chitosan‐induced mitochondrial stress in DCs can result in increased mitochondrial ROS and cytoplasmic DNA, leading to the activation of cGAS‐STING‐IFN‐I axis and the maturation of adjacent DCs.^[^ ^71^ ^]^ While there have been few studies reporting the immunomodulatory activity of dextran and its derivatives, our research found that oxidized dextran showed a comparable immunostimulatory effect to CH. The underlying mechanisms remain to be further explored.

In conclusion, CH‐OD hydrogel offers a flexible platform for tumor therapy due to its controlled drug‐release properties and intrinsic immunostimulatory capability. Our findings suggest that the local application of CH‐OD‐SSZ has the potential to sensitize tumors to immunotherapy and augment antitumor immunity in a safe and effective manner. In the mouse model of advanced‐stage HCC with established ascites, CH‐OD‐SSZ exhibited potent therapeutic efficacy and long‐term protective effects.

## 4 Conclusion

In summary, we synthesized an injectable polysaccharide hydrogel, CH‐OD with promising immunostimulatory effects for the local delivery of SSZ to potentiate treatment for HCC‐related ascites. The encapsulated SSZ was gradually released to kill tumor cells directly and induce immunogenic ferroptosis to provoke an antitumor immune response. Both in vitro and in vivo, CH‐OD‐SSZ hydrogel could promote the activation of DCs and repolarize TAMs towards an M1‐like phenotype, thus remedying the TIME. The results demonstrated that intraperitoneal administration of CH‐OD‐SSZ hydrogel achieved a 25% complete response rate in a mouse model of HCC ascites. The combination of anti‐PD‐1 with CH‐OD‐SSZ hydrogel further promoted the therapeutic effects, leading to >50% regression of ascites and the establishment of long‐term protective immune memory. Overall, this injectable CH‐OD‐SSZ hydrogel shows promise in combination with immunotherapy for increasing treatment response in advanced HCC with peritoneal dissemination and malignant ascites.

## 5 Experimental Section

### Reagents

Ferrostatin‐1 (S7243), deferoxamine (S5742), and sulfasalazine (S1576) were purchased from Selleck. *β*‐Mercaptoethanol (07604) was purchased from Sigma‐Aldrich. H2DCFDA (D399) and BODIPY 581/591 C11 (D3861) were purchased from Invitrogen. Dextran (*M*_n_ = 250 kDa) and chitosan (low molecular weight, deacetylated chitin) were purchased from Sigma‐Aldrich (Shanghai, P. R. China). Sodium periodate (NaIO_4_, 99.5%) was obtained from Rhawn reagent (Shanghai, P. R. China). Hydroxylamine hydrochloric acid and methyl orange were purchased from Aladdin (Shanghai, P. R. China). Sodium hydroxide, diethylene glycol, and acetone were obtained from Xilong Scientific (Shantou, P. R. China). Dimethyl sulfoxide (DMSO) was obtained from Sigma‐Aldrich (Shanghai, P. R. China).

### Preparation of Oxidized Dextran

A total of 1.11 g of NaIO_4_ (1.0 equiv., dissolved in 20 mL of water) was added dropwise to 100 mL of dextran solution (5% w/v, 6.0 equiv.) under dark conditions. The reaction system was stirred for 12 h at 25 °C in the dark. An equimolar amount of diethylene glycol was added to quench the unreacted NaIO_4_. Further, the mixture was dialyzed against water in a dialysis membrane (molecular weight cut‐off (MWCO) = 14 kDa) for three days, frozen overnight at −80 °C, and then freeze‐dried using a lyophilizer (Christ, Germany) to obtain OD (yield, 80%). The oxidation degree of OD was detected by hydroxylamine hydrochloric acid titration. Briefly, 0.1 g OD was added to methyl orange/hydroxylamine hydrochloride solution. Subsequently, the above solution was titrated with 0.1 mol L^−1^ NaOH until the solution color changed from red to yellow. The oxidation degree of OD was calculated:

(1) $$\mathrm{oxidation}\;\mathrm{degree}\;\left(\%\right)=\frac{V_{\mathrm{NaOH}}\times {\mathrm{mol}}^{-1}\times {\mathrm{Mw}}_{\mathrm{dextran}}}{1000\times m_{\mathrm{dextran}}}\times 100$$

where *V*_NaOH_, Mw_dextran_, and *m*_dextran_ were the volume of NaOH used for titration, the molecular weight of dextran monomer, and the weight of dextran, respectively.

### Preparation of Chitosan Hydrochloride

CH was prepared by the method of Austin and Sennett.^[^ ^72^ ^]^ Chitosan (5.0 g) dispersed in 50 mL of 60% ethanolic HCl was kept stirring magnetically for 3 h at 25 °C. The hydrochloride salt thus formed was then filtered off, washed extensively with acetone‐water mixture (*v*/*v* = 3:1), and dialyzed against water until the dialysate reached neutral pH. The product was then freeze‐dried and stored at 4 °C until use.

### Preparation of CH‐OD Hydrogel

CH solution of 2.0% (w/v) and OD solution of 5.0% (w/v) in PBS were prepared, respectively. To elucidate the influence of the feed ratio of CH and OD on the properties of hydrogels, 2.0% CH solution was mixed with 5.0% OD solution in different volume ratios including 1:1.3, 1:2, 1:3, and 1:4, which corresponded to the ratio between amino group and aldehyde group of 1.5:1, 1:1, 1:1.5, and 1:2, respectively. The final concentration of hydrogel was kept at 3.5% (w/v). The mixed solutions were vortexed for 5 s to form the hydrogel.

### Preparation of CH‐OD‐SSZ Hydrogel

To prepare the CH‐OD‐SSZ hydrogel, a stock solution of SSZ (200 mg mL^−1^) was prepared using DMSO as the solvent. 80 µL SZZ solution was added to 420 µL OD solution of 6.4% (w/v) in PBS, which was mixed with 500 µL CH solution of 1.6% (w/v) in PBS. The mixed 1.0 mL solution was vortexed for 5 s to obtain SSZ‐loaded CH‐OD hydrogel. For animal experiments, a stock solution of SSZ (250 mg mL^−1^) was prepared in DMSO, and 80 µL of this solution was used for the preparation of 1.0 mL CH‐OD‐SSZ hydrogel with a loading SSZ concentration of 50 mm.

### Rheological Test

TA rheometer (DHR‐2) was used to evaluate the rheological properties of the hydrogels. The storage modulus (*G'*) and loss modulus (*G“”*) were investigated by placing the mixture between parallel plates with a 20 mm diameter and a 1.0 mm gap. 0.3 mL hydrogel (CH‐OD or CH‐OD‐SSZ) precursor solution was placed between parallel plates, respectively. The experiment was performed using a time sweep test at 37 °C with a frequency of 10 rads^−1^ and 1% strain. The effect of shear rate on viscosity was observed in the flow sweep measurement with a shear rate range from 0.01 to 100 s^−1^. Flow peak hold measurements with a fixed shear rate of 0.1 s^−1^ was carried out to observe the process of hydrogel reaching a steady state. All experiments were performed at 37 °C utilizing a 20 mm parallel plate with a 1.0 mm gap.

### Swelling Test

To investigate the swelling property of the hydrogel, the prepared hydrogel samples were lyophilized and then immersed in PBS at 37 °C until completely swelling. First, the lyophilized hydrogels were weighed as *M*_0_ before immersion. After soaking in PBS for different times, the hydrogels were removed from PBS and weighed as *M_x_*. The swelling ratio of hydrogels was calculated as *S*% *=* (*M_x_−M*_0_) */M*_0_ *×*100.

### Hydrogel Degradation In Vitro and In Vivo

To assess in vitro degradation of the hydrogels, 0.5 mL of hydrogels was added to the bottom of a 5 mL cap glass bottle, followed by 2 mL PBS with or without 5 U mL^−1^ protease K. The capped glass bottles were placed on a 37 °C shaker so that the hydrogels were in full contact with the dissolving medium. The mass of the remaining hydrogels was weighed daily and its degradation quality was calculated. The degradation curve was drawn according to the data. Then the degradation of the hydrogels was tested in vivo. After mice were anesthetized, 50 µL hydrogel was injected subcutaneously into the right flank of mice, and the skin bulge was observed and photographed every seven days.

### Scanning Electron Microscopy (SEM)

The surface and internal morphology of OD‐CH hydrogels were examined by SEM. Lyophilized hydrogels were cut using a razor blade to expose the inner region, placed on double‐sided tape, sputter coated with gold, and examined in the microscope (ZEISS SIGMA HD) for internal structure.

### In Vitro Drug Release Study

The in vitro release experiments of SSZ from CH‐OD‐SSZ hydrogel depots were performed at 37 °C. 10 µL CH‐OD‐SSZ hydrogel was placed in 12‐well plates and 1 mL PBS was added as dissolved media. Then the PBS was collected at 4, 8, 12, 24, 36, 48, and 72 h respectively, and the content of SSZ was determined and analyzed by high‐performance liquid chromatography (HPLC, 1100 LC/MSD Trap, Agilent, USA).

### Bioinformatics Analysis

The information on liver hepatocellular carcinoma (LIHC) and adjacent non‐tumorous samples was obtained from The Cancer Genome Atlas (TCGA) database and then analyzed by R language (Version 4.0). The expression of SLC7A11 in different stages and the correlation with immune infiltration were analyzed by GEPIA 2.0 database (http://gepia2.cancer‐pku.cn/#index).

### Cell Line and Cell Culture

The H22 murine hepatoma cell line was purchased from the China Center for Type Culture Collection (Wuhan) and cultured with DMEM (Gibco) containing 10% fetal bovine serum (FBS, Gibco) and 1% penicillin/streptomycin at 37 °C atmosphere with 5% CO_2_.

### Colony Formation Assay

Poly‐*d*‐lysine solution (PDL, ThermoFisher Scientific) was added to the 6‐well cell culture plate (1 mL per well) and incubated at 37 °C for 1 h. Then, the solution was removed and the plate was washed with sterile water three times. After the PDL‐coated plate was dry, H22 cells were seeded into each well (500 cells per well) and treated with PBS, SSZ (0.4 mm), CH‐OD (10 µL), or CH‐OD‐SSZ (10 µL) for 48 h. When colonies had more than 50 cells, the cells were fixed with 4% formaldehyde for 20 mins, stained with 2% crystal violet for 2 h, and carefully washed with water. The stained cells were then photographed, and the number of clones was counted.

### Cell Viability Experiment

H22 cells were plated into 96‐well plates (1×105 cells per well) and allowed to grow for 24 h. Then cells were treated with PBS, SSZ (0.4 mm), CH‐OD (10 µL), and CH‐OD‐SSZ (10 µL) for 24 h. Then CCK‐8 assay solution (BS350B, Biosharp) at a 1:20 dilution with serum‐free DMEM (100 µL) was added to each well and the absorbance at 450 nm was measured after a further 1 h incubation.

### Intracellular ROS Measurement

For the detection of intracellular ROS level, H22 cells were stained with 10 µm H2DCFDA for 30 min at 37 °C, then washed three times with PBS, suspended in 200 µL of fresh PBS, and analyzed immediately using a flow cytometer.

### Intracellular GSH Measurement

Intracellular GSH levels were measured using Intracellular glutathione (GSH) Detection Assay Kit (Abcam) and the fluorescence intensity was detected by flow cytometry at *E_x_*/*E_m_* = 490/520 nm.

### ATP Release Assay

To measure the release of intracellular ATP, cell culture supernatants were collected and the concentrations of ATP were measured using luciferin‐based ENLITEN ATP Assay (Promega) kits in accordance with the manufacturer's protocol.

### CRT Assay

Cells were seeded in a 12‐well cell culture plate and incubated with SSZ, CH‐OD, and CH‐OD‐SSZ hydrogel for 24 h. Cells were fixed in formaldehyde (1% in PBS) and incubated with the anti‐CRT antibody for 30 min, followed by incubation with fluorescein‐conjugated AffiniPure goat anti‐rabbit IgG for 30 min. Then, cells were washed and resuspended with 100 µL PBS for flow cytometry analysis.

### Transmission Electron Microscopy

H22 cells treated with CH‐OD‐SSZ hydrogel were fixed with 1% formaldehyde and 2.5% glutaraldehyde in 0.1 m Sodium cacodylate buffer for 1 h at room temperature, washed 3 times in cacodylate buffer, then post‐fixed in 1% osmium tetroxide in H_2_O for 1 h and washed in H_2_O for 3 times. Next, samples were dehydrated in graded ethanol concentrations and propylene oxide, and embedded in Spurr's resin. Ultrathin frozen sections were then prepared and transferred to copper grids, stained with 0.75% uranyl acetate or 1% lead citrate, and observed by TEM (HITACHI HT7700 120v).

### Generation of BMDMs and BMDCs

Bone marrow cells were obtained from femurs and tibiae of 6‐week‐old BALB/c male mice. For the generation of BMDMs, after the depletion of red blood cells (RBCs) using RBC lysis buffer, bone marrow cells were cultured in RPMI 1640 medium supplemented with 10% FBS, 1% penicillin/streptomycin, and recombinant mouse M‐CSF (20 ng mL^−1^, PeproTech) for 7 days. The culture medium was replaced on the 3rd and 5th day respectively. On the 6th day, BMDMs were stimulated with IL‐4 (20 ng mL^−1^, PeproTech) for 24 h to polarize macrophages to M2 phenotype. For BMDCs, bone marrow cells were supplied with recombinant mouse GM‐CSF (20 ng mL^−1^, PeproTech) for 7 days and non‐adherent cells were collected for further experiments.

### Animal Studies

Female BALB/c wild‐type mice (6‐week‐old) were purchased from HBCDC (Wuhan, China). To establish the mice model of H22 hepatocarcinoma ascites, 2×10^5^ H22‐luc cells were implanted intraperitoneally (i.p.). Three days later, bioluminescent imaging was used to confirm the formation of ascites, and mice were randomly allocated into different groups. PBS (100 µL), SSZ (2 mg per mouse in 100 µL PBS), CH‐OD (100 µL), and CH‐OD‐SSZ (containing 2 mg SSZ, 100 µL) hydrogel were administered i.p. on days 4, 7, and 10. For PD‐1 blockade, an anti‐PD‐1 mAb (10 mg kg^−1^) was injected i.p. every other day for four times. Tumor burden was evaluated on day 12 by in vivo imaging. Mice were maintained in a specific pathogen‐free facility, and all experimental protocols were approved by the Medical Ethics Committee of Tongji Medical College, Huazhong University of Science and Technology (3132).

### Flow Cytometry Analysis

Ascites fluid was collected from the peritoneal cavity of the mice and centrifuged at 500 g for 5 min at 4 °C. After lysis of RBCs and twice washes with ice‐cold PBS, cells were resuspended with 100 µL PBS at the concentration of 1 × 10^6^ cells mL^−1^, and incubated with Zombie Violet Viability kit to label dead cells. For analysis of T cells, samples were stimulated with 1 mg mL^−1^ ionomycin (Abcam), 50 ng mL^−1^ phorbol 12‐myristate 13‐acetate, and 1.5 µg mL^−1^ monensin for 6 h at 37 °C, and then stained with anti‐CD45, anti‐CD3*ε*, anti‐CD4, and anti‐CD8a fluorescent antibodies. After fixation and permeabilization, cells were further stained with anti‐IFN‐*γ* and anti‐Foxp3 antibodies. For analysis of macrophages and DCs, antibodies used were as follows: anti‐CD45, anti‐CD11b, anti‐F4/80, anti‐CD11c, anti‐CD86, anti‐I‐A^d^, and were stained after fixation and permeabilization. All fluorescent antibodies were incubated on ice for 30 min in the dark and were against mouse antigens (all from Biolegend).

### Bioluminescence Imaging

For in vivo bioluminescence imaging, mice were anesthetized with 150 µL 1% pentobarbital sodium and then intraperitoneally injected with 150 mg kg^−1^ firefly luciferin (Sigma‐Aldrich; CAS: 103404‐75‐7). After 10 min, mice were imaged by the Bruker In vivo MS FX PRO Imager. The luminescent images were acquired with a 3‐min exposure time, and X‐ray photographs were taken with a 30‐s exposure time. The average signal intensities within a circular region of interest (ROI) were quantified.

### RNA‐seq Analysis

RNA‐seq was performed by Seqhealth Technology Co., LTD (Wuhan, China). Raw sequencing data were filtered by Trimmomatic (version 0.36) to obtain clean data, which were then mapped using STRA software (version 2.5.3a). Reads mapped to the exon region of each gene were counted by using featureCounts (Subread‐1.5.1; Bioconductor). And then RPKMs were calculated. Differential gene expression between the two groups was identified by using edgeR package (version 3.12.1). A fold‐change cutoff of 2 and a p‐value cutoff of 0.05 were used to judge the statistical significance of gene expression differences. KEGG enrichment analysis was implemented by KOBAS software (version: 2.1.1) to judge statistically significant enrichment.

### Statistical Analysis

All the data were presented as the mean ± SEM from at least three replicates. For comparisons of the two groups, a two‐tailed unpaired t‐test was performed. For comparisons of three or more groups, one‐way ANOVA was performed with Tukey's multiple comparisons test. One‐way ANOVA was also used for comparing two groups in multi‐group comparisons. Tumor growth was assessed by two‐way ANOVA. Statistical analysis was performed with GraphPad Prism 9.0 software (GraphPad Software, Inc.). *P* < 0.05 were considered to be statistically significant. Data are presented as means ± SEM. *P < 0.05; **P < 0.01; ***P < 0.001, and n.s., not significant.

## Conflict of Interest

The authors declare no conflict of interest.

## Supporting information

Supporting Information

## Data Availability

The data that support the findings of this study are available from the corresponding author upon reasonable request.
