# Supplementary material for: Ferroptosis‐Enhanced Immunotherapy with an Injectable Dextran‐Chitosan Hydrogel for the Treatment of Malignant Ascites in Hepatocellular Carcinoma
Source: Adv Sci (Weinh). 2023 May 3;10(20):2300517. doi: 10.1002/advs.202300517 (PMC10369279; doi:10.1002/advs.202300517)
Supplement: Supplementary file 1 — Supporting Information [file ADVS-10-2300517-s001.pdf]

ADVANCED SCIENCE
Open Access

Supporting Information

for *Adv. Sci.*, DOI 10.1002/advs.202300517

Ferroptosis-Enhanced Immunotherapy with an Injectable Dextran-Chitosan Hydrogel for the Treatment of Malignant Ascites in Hepatocellular Carcinoma

Jingshu Meng, Xiao Yang, Jing Huang, Zhan Tuo, Yan Hu, Zhiyun Liao, Yu Tian, Suke Deng, Yue Deng, Zhiyuan Zhou, Jonathan F. Lovell, Honglin Jin, Yang Liu* and Kunyu Yang**

Supplementary information

Ferroptosis-Enhanced Immunotherapy with an Injectable Dextran-Chitosan Hydrogel for the Treatment of Malignant Ascites in Hepatocellular Carcinoma

Jingshu Meng[1], Xiao Yang[1], Jing Huang[1], Zhan Tuo, Yan Hu, Zhiyun Liao, Yu Tian, Suke Deng, Yue Deng, Zhiyuan Zhou, Jonathan F. Lovell, Honglin Jin,[*] Yang Liu,[*] and Kunyu Yang[*]

J. Meng, X. Yang, Z. Tuo, Y. Hu, Z. Liao, Y. Tian, S. Deng, Y. Deng, Z. Zhou, K. Yang

Cancer Center, Union Hospital, Tongji Medical College, Huazhong University of Science and Technology, Wuhan 430022, China

J. Huang, H. Jin

College of Biomedicine and Health and College of Life Science and Technology, Huazhong Agricultural University, Wuhan, 430070, China

J. F. Lovell

Department of Chemical and Biological Engineering, University at Buffalo, State University of New York. Buffalo, New York 14260, USA

Y. Liu

Key Laboratory of Polymer Ecomaterials, Changchun Institute of Applied Chemistry, Chinese Academy of Sciences, 5625 Renmin Street, Changchun 130022, P. R. China

[1] These authors contributed equally to this work.

[*]Corresponding authors

H. Jin, E-mail address: jin@hust.edu.cn; Y. Liu, E-mail address: yangl@ciac.ac.cn;

K. Yang, E-mail address: yangkunyu@hust.edu.cn.

Keywords: cancer immunotherapy, ferroptosis, hydrogel, malignant ascites, tumor microenvironment

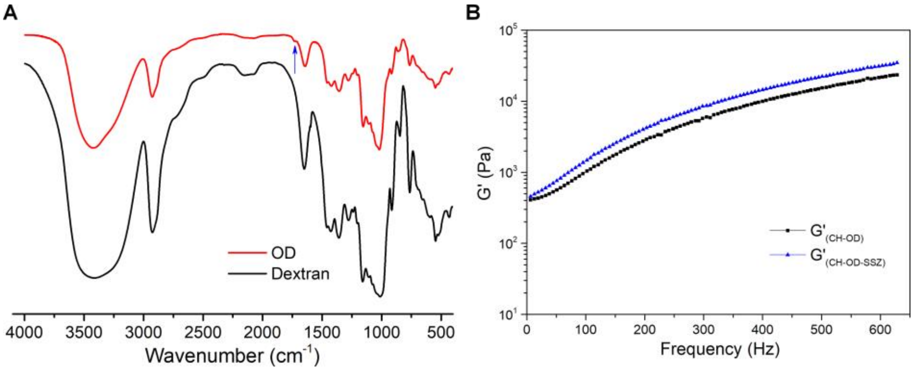

Figure S1. A) Fourier transform infrared spectra of OD and dextran. B) Frequency dependence of storage modulus (*G′*) for a 3.5% CH-OD and CH-OD-SSZ hydrogel.

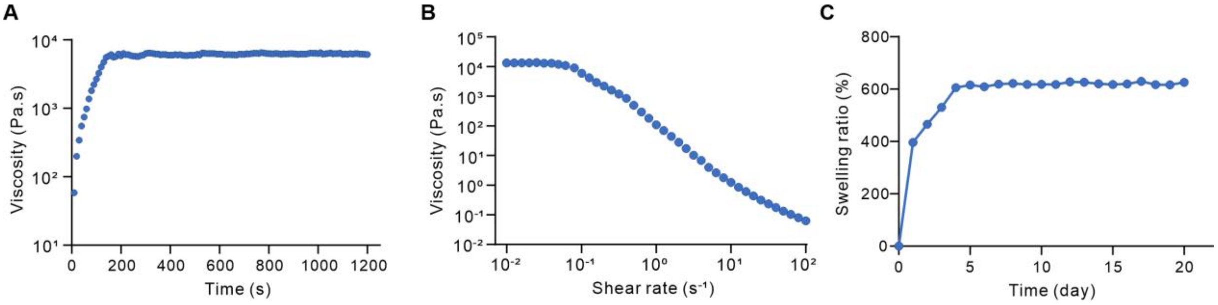

Figure S2. The rheological characteristics and swelling ratio of the hydrogels. A) Flow peak hold measurements with a fixed shear rate of 0.1 s^{-1}. B) Steady shear rheology of the hydrogel. C) Swelling ratio of the hydrogel.

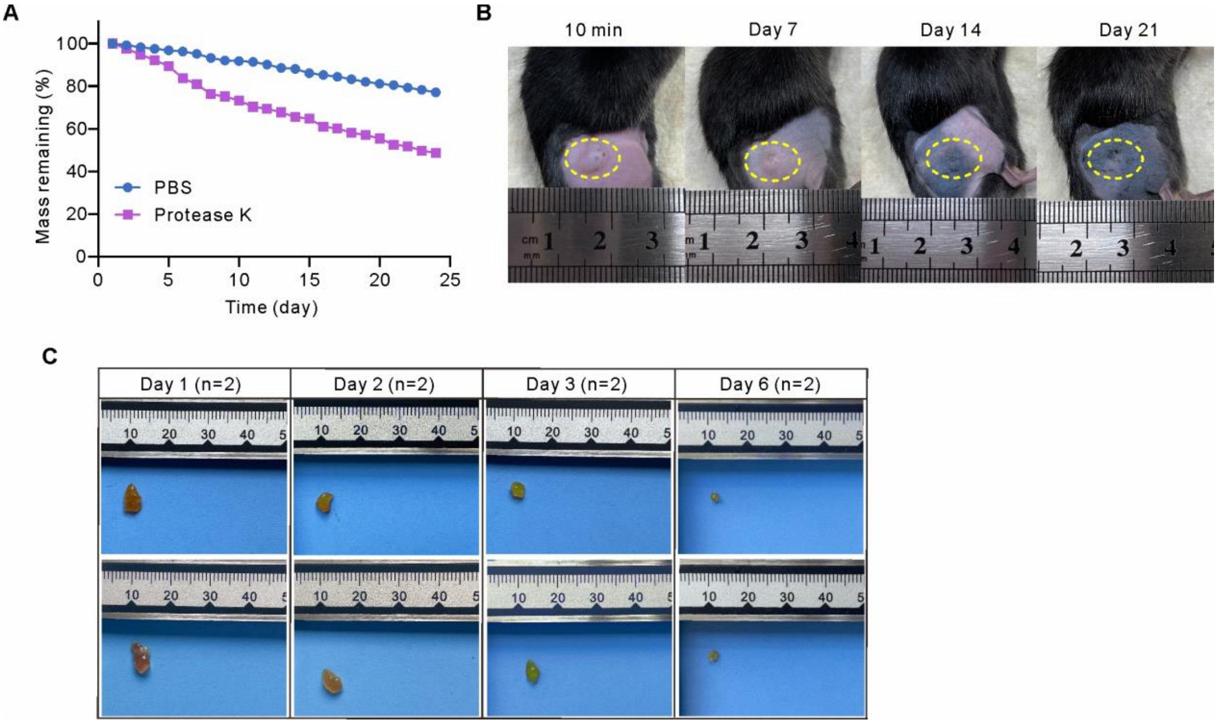

Figure S3. Degradation of hydrogels *in vitro* and *in vivo*. A) Percentage of remaining mass of hydrogel *in vitro*. B) *In vivo* degradation of hydrogel. C) Remained CH-OD-SSZ hydrogels in mice with formed malignant ascites at day 1, 2, 3 and 6 after peritoneal injection.

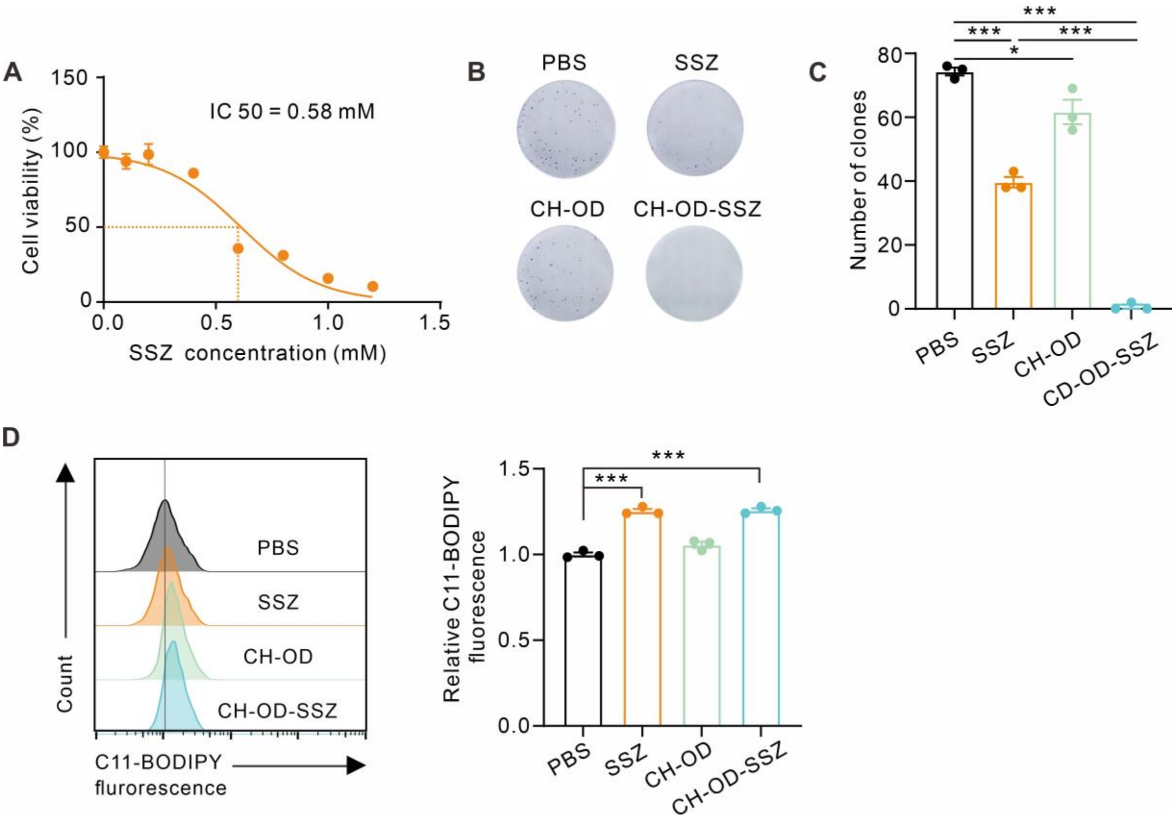

Figure S4. In vitro killing effect of different formulations. A) The IC50 value of SSZ in H22 cells (24 h). B) Representative images showing cell clones of H22 cells treated with PBS, SSZ, CH-OD or CH-OD-SSZ hydrogel. C) Quantification of colony formation analysis. D) Lipid peroxidation was assessed by flow cytometry using BODIPY 581/591 C11 fluorescent probe. The indicated results represent the means ± SEM of three independent experiments. * $P < 0.05$, *** $P < 0.001$, analyzed by one-way ANOVA.

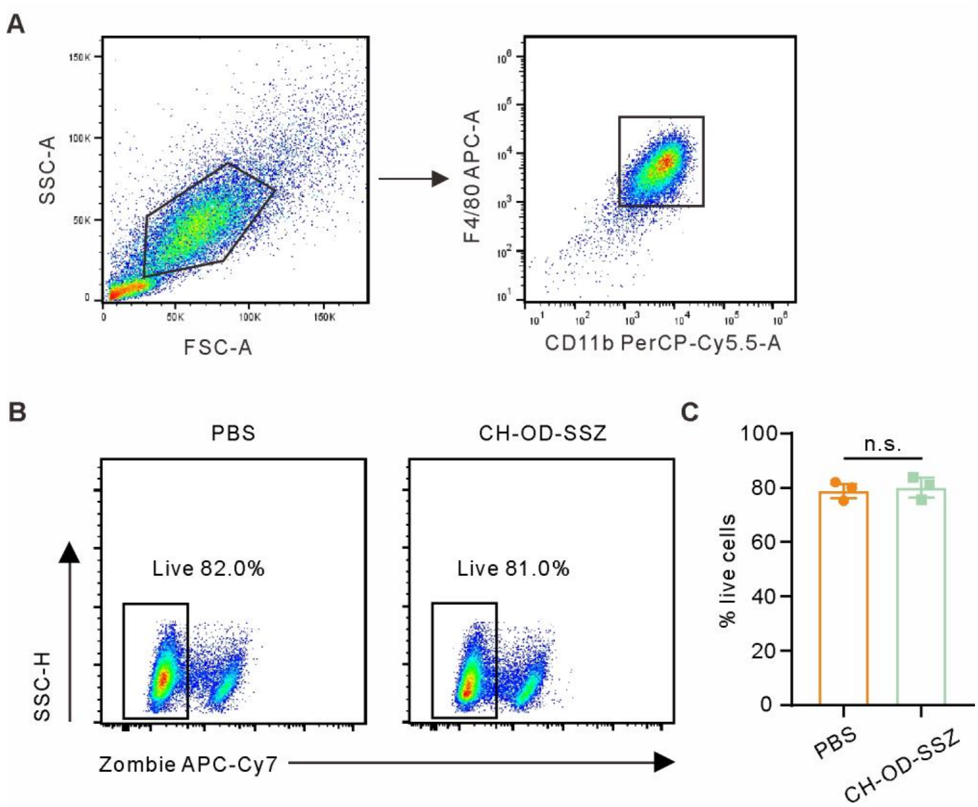

Figure S5. A) Gating strategy for the flow cytometry analysis of BMDMs. BMDMs were characterized as $CD11b^{+}F4/80^{+}$ cells. B,C) Cell viability of BMDMs treated with CH-OD-SSZ for 24 h was detected by Zombie dye staining with flow cytometry. The indicated results represent the means ± SEM of three replicates, n.s., not significant.

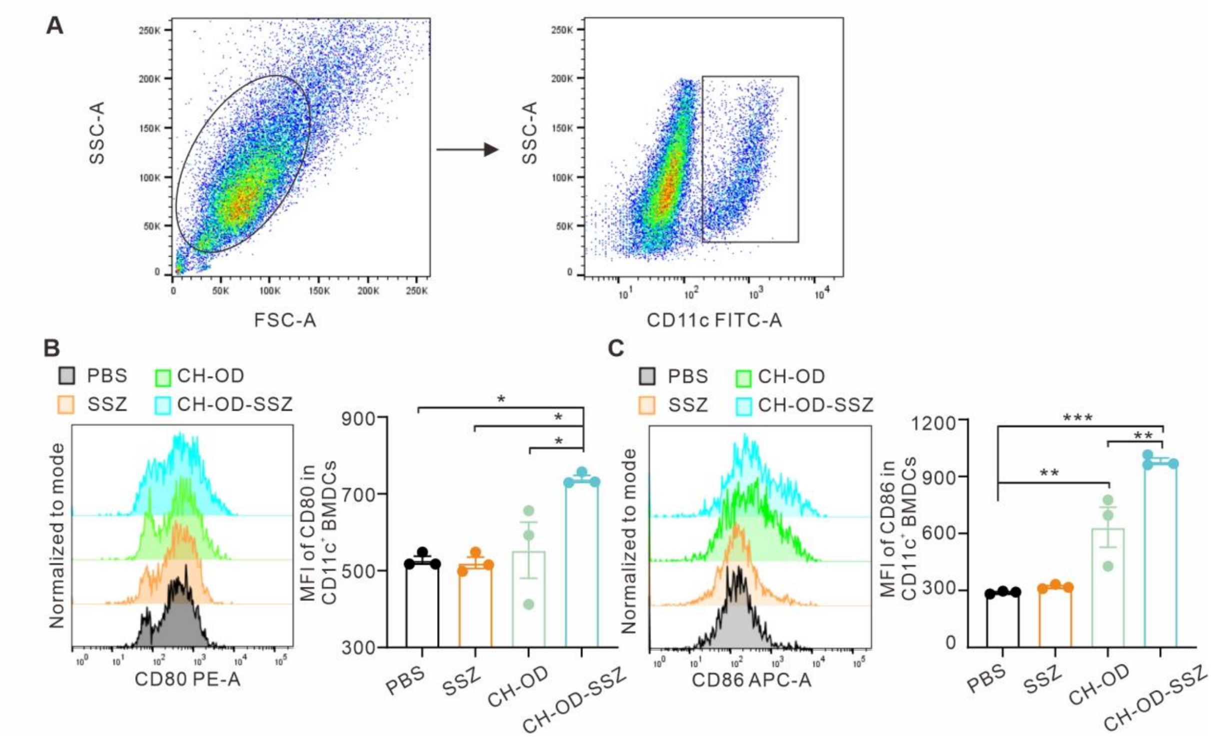

Figure S6. A) Gating strategy for the flow cytometry analysis of BMDCs. BMDCs were characterized as CD11c$^+$ cells. B,C) Flow cytometry analysis for the expression of CD80 and CD86 in BMDCs. BMDCs were cocultured with PBS, SSZ, CH-OD or CH-OD-SSZ-treated H22 cells for 24 h. The indicated results represent the means ± SEM of three independent experiments. * P < 0.05, ** P < 0.01, *** P < 0.001, analyzed by one-way ANOVA.

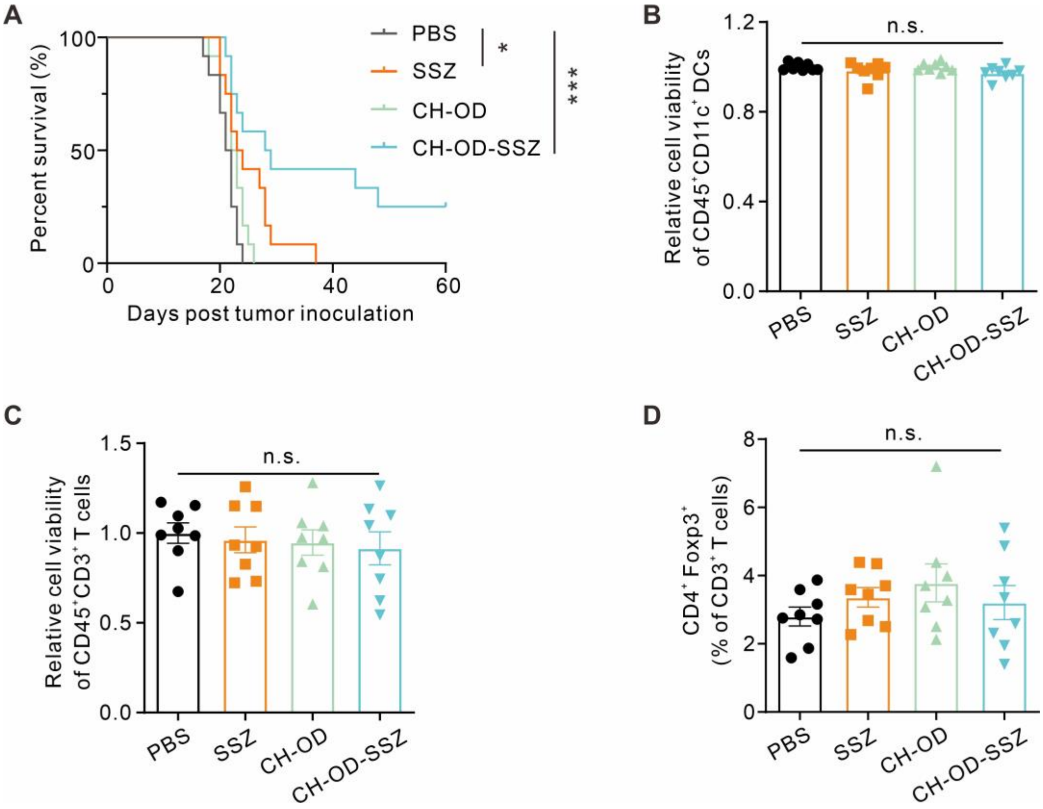

Figure S7. A) Survival curves of mice with H22-luc malignant ascites in different treatment groups (n = 12 mice per group). Day 0 indicates mice random grouping; Day 1 indicates treatment beginning. B,C) Cell viability of DCs ($CD45^+CD11c^+$) (B) and T cells ($CD45^+CD3^+$) (C) within ascites of each group were determined according to Zombie dye negative staining (n=8 mice per group). D) Percentages of Tregs ($CD4^+Foxp3^+$) within ascites of each group (n=8 mice per group). The indicated results represent the means ± SEM of three independent experiments. * $P < 0.05$, *** $P < 0.001$, n.s., not significant.

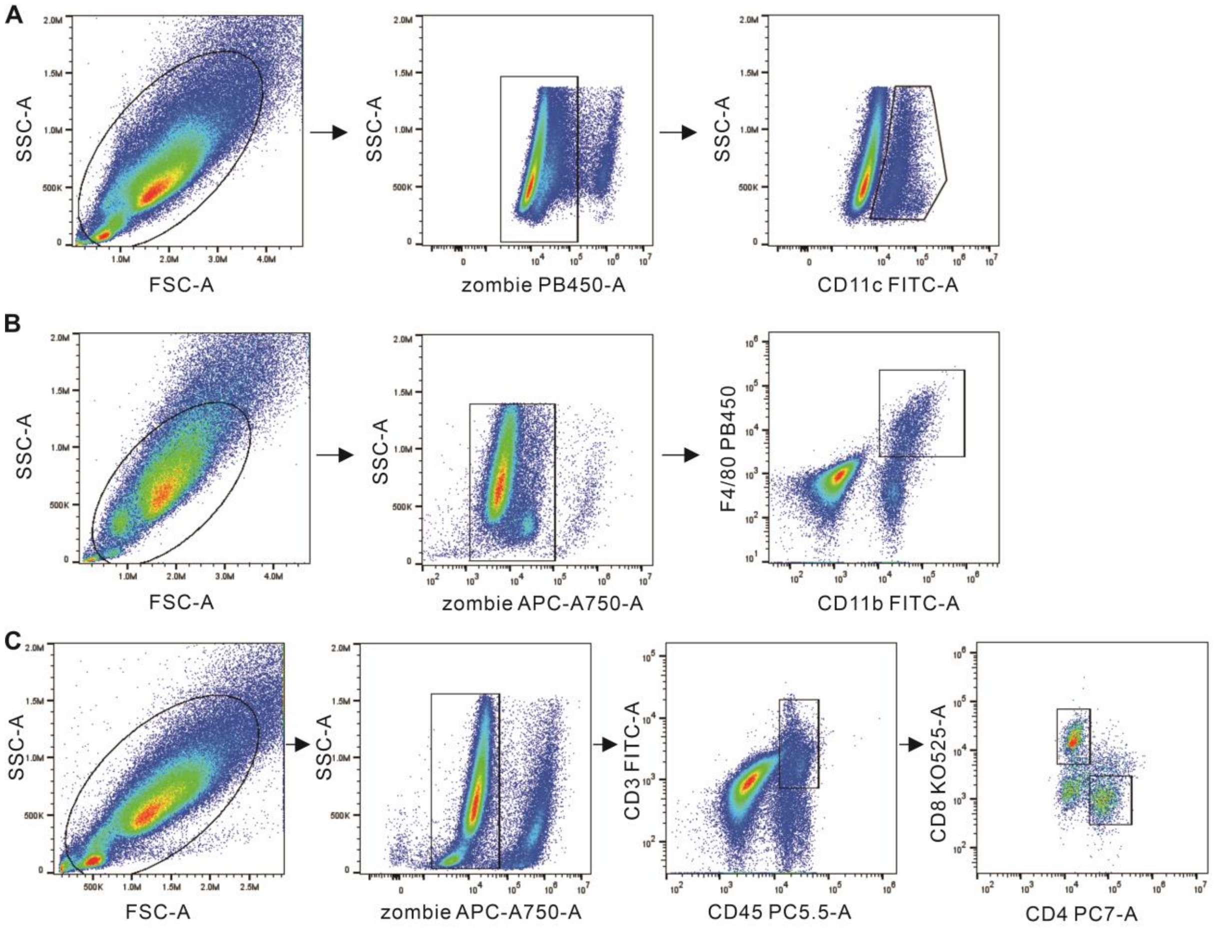

Figure S8. Gating strategy for the analysis of immune cells within the peritoneal lavage. A) DCs, B) macrophages, C) CD4$^+$ T cells and CD8$^+$ T cells.

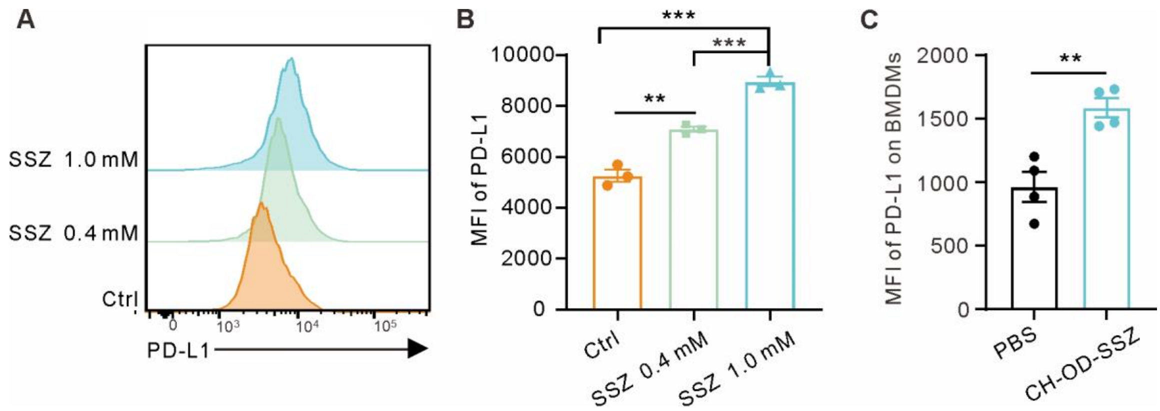

Figure S9. A,B) The expression of PD-L1 on the surface of H22 cells treated with SSZ (0.4 and 1 mM) for 24 h was detected by flow cytometry. C) The expression of PD-L1 on the surface of BMDMs treated with PBS or CH-OD-SSZ hydrogel for 24 h was detected by flow cytometry. The indicated results represent the means ± SEM of three replicates, ** $P < 0.01$, *** $P < 0.001$.

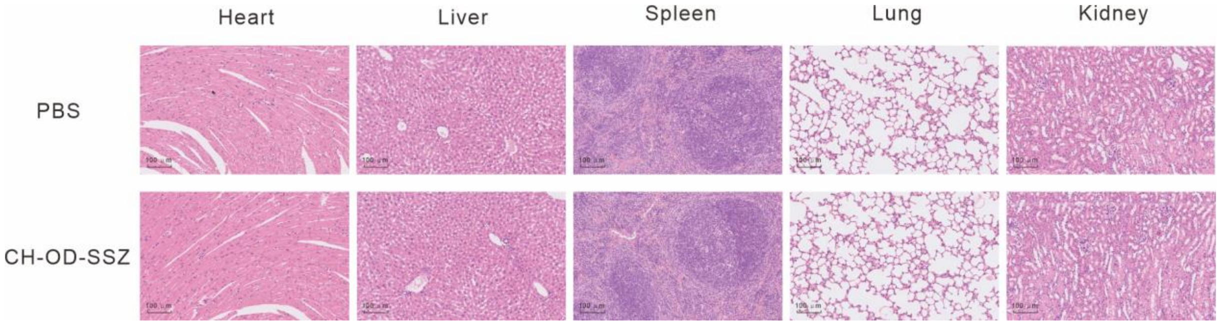

Figure S10. Representative images of HE staining of the heart, liver, spleen, lung and kidney of mice receiving PBS or CH-OD-SSZ hydrogels treatment. Scale bar, 100μm.
